# Assessment of Psychosocial Stress and Mental Health Disorders in Parents and Their Children in Early Childhood: Cross-Sectional Results from the SKKIPPI Cohort Study

**DOI:** 10.3390/children11080920

**Published:** 2024-07-30

**Authors:** Julia Fricke, Marie Bolster, Katja Icke, Natalja Lisewski, Lars Kuchinke, Christiane Ludwig-Körner, Franziska Schlensog-Schuster, Thomas Reinhold, Anne Berghöfer, Stephanie Roll, Thomas Keil

**Affiliations:** 1 Institute of Social Medicine, Epidemiology and Health Economics, Charité—Universitätsmedizin Berlin, Corporate Member of Freie Universität Berlin and Humboldt-Universität zu Berlin, 10117 Berlin, Germany; julia.fricke@lkbh.de (J.F.); marie.bolster@charite.de (M.B.); katja.icke@charite.de (K.I.); thomas.reinhold@charite.de (T.R.); stephanie.roll@charite.de (S.R.); thomas.keil@charite.de (T.K.); 2 Unit for Municipal Health Strategies for the City of Freiburg and the District of Breisgau-Hochschwarzwald, 79104 Freiburg, Germany; 3 International Psychoanalytic University, 10555 Berlin, Germany; lars.kuchinke@ipu-berlin.de (L.K.); christiane.ludwig-koerner@ipu-berlin.de (C.L.-K.); 4 University Hospital of Child and Adolescent Psychiatry and Psychotherapy, University of Bern, 3000 Bern, Switzerland; franziska.schlensogschuster@upd.ch; 5 Institute for Complementary and Integrative Medicine, University Hospital Zurich, University of Zurich, 8091 Zurich, Switzerland; 6 Institute for Clinical Epidemiology and Biometry, University of Wuerzburg, 97070 Wuerzburg, Germany; 7 State Institute of Health, Bavarian Health and Food Safety Authority, 97688 Bad Kissingen, Germany

**Keywords:** early childhood, postnatal, parents, stressors, mental health, regulatory problems, mother-child attachment

## Abstract

Background/Objectives: Early childhood can be a stressful period for families with a significant impact on parents’ mental health, the child’s healthy development, and the development of a secure mother–child attachment. The goal of the cross-sectional study part of SKKIPPI was to assess the occurrence of psychosocial stress and mental health disorders in parents as well as in their offspring in early childhood in three German regions. Methods: Based on random samples from three residents’ registration offices, parents with infants aged up to 12 months were invited to participate. An online screening questionnaire was developed in four languages to assess common psychosocial stressors and mental health problems of parents with small children. Results: The study enrolled 4984 mothers and 962 fathers. The most common potential psychosocial stressors were professional problems (mothers 22%, fathers 33%), lack of social support (20%, 14%), and severe, negative experiences in childhood (22%, 16%). Obsessive–compulsive thoughts (21%, 16%) and depressive (9%, 9%) and anxiety symptoms (11%, 7%) were the most frequently reported mental health problems by both parents. Regulatory problems of the child were reported by between 1.5% and 5.1% of parents. Conclusions: The study showed that a substantial proportion of parents are burdened by psychosocial problems and suffer from mental health problems in the first years after the birth of their children. Early preventive and low-threshold support measures should be available in the health and social care system. Low-threshold questionnaires, which cover a wide range of possible stress factors, should be further developed for the practical healthcare of this group of people.

## 1. Introduction

The first years after the birth of a child can come with a number of stressors for the parents. This can lead to impairments in mental health not only in parents themselves, but also in their children. Children of psychosocially stressed mothers and fathers are at high risk of social, emotional, and cognitive problems [1] because the development of a positive mother–child relationship is considered an essential resilience factor for child development. Maternal sensitivity in dealing with the infant is considered a key variable in mother–child interaction and child attachment development [2,3,4]. A good parent–child relationship and a secure infant attachment have been identified as moderators of a child’s healthy psychological and physical development [4].

Common factors for a strained parent–child relationship are stress, psychological burden, and mental disorders. Such factors limit the parental ability to sensitively perceive, correctly interpret, and appropriately respond to the child’s signals [2,5]. Furthermore, these stress factors influence the parents’ reflexive ability to empathize with their child’s feelings and needs and to differentiate these from their own inner states [6], thus establishing negative interaction cycles that have a considerable impact on the development of regulatory disorders, insecure attachment patterns, child behavioral problems, and psychopathologies [7,8,9]. Affected parents and children should not be considered separately. For example, up to 18% of infants and young children also show psychological and/or psychosomatic symptoms in the form of early childhood regulatory disorders [10]. Also, the additional burden of illness and social isolation, which has affected many families during the coronavirus pandemic, has already had an impact on mother–child bonding and the frequency and severity of regulatory disorders in infants [11].

The occurrence of mental health disorders in mothers, especially postnatal depression and anxiety, has been extensively studied worldwide. Studies on the prevalence of mental health disorders in mothers are showing large variations, with global prevalence estimates of 18% for postnatal depression and 15% and 19%, respectively, for postnatal anxiety symptoms and anxiety disorders [12,13,14]. Children of mothers with postnatal mental illness seem to have an increased risk of developing their own psychological problems. Martini and colleagues showed that in infants, regulatory disorders like sleeping or feeding problems or excessive crying were associated with maternal mental health problems [15]. Population-based studies from Denmark and Australia revealed prevalence rates of regulatory problems of up to 21% in the first two years of life of the children [16,17]. Postnatal mental health in fathers has not been a focus of research for a long time, but in the last two decades there has been an increase in the number of studies devoted to this disorder. A meta-analysis by Cameron and colleagues assessed a prevalence rate of 8.4% for paternal prenatal and postpartum depression [18]. In Germany, prevalence data on mental health problems in parents and their infants in the first years of life are mostly derived from hospital- or outpatient-based studies, often including only mothers and not fathers [15,19]. Under the impact of the COVID-19 pandemic, the stress of young parents has increasingly come into focus. The German Coronababy study investigated stress and mental disorders of parents and infants as well as regulatory disorders of infants during the pandemic in Bavaria and found inadequate support for those affected during the pandemic [20,21].

There are several well-known determinants for maternal mental health problems in the postpartum period and beyond, for example, an unplanned pregnancy or a lack of social support [22,23,24]. In a German study assessing psychosocial stressors in families with infants and toddlers, 20% of the parents reported lack of family and social support, and 21% reported an unplanned pregnancy [25]. Given the high percentages and the well-established associations between parental stressors and mental health in parents and their children in their first years of life, data from population-based studies assessing mental health problems in parents and children are needed. Population-based data could provide an overall picture of the mental health status of parents and their young children and identify potential gaps in the healthcare and social system in Germany. 

The presented cross-sectional study was established to assess the occurrence of psychosocial stress and mental health disorders in parents and their offspring in early childhood [26]. The study is part of the multi-component project SKKIPPI (Evaluation der Eltern-**S**äugling-**K**lein**KI**nd-**P**sychotherapie mittels **P**rävalenz- und **I**nterventionsstudien/ Evaluation of parent-infant psychotherapy using prevalence and intervention studies) with randomized intervention as well as qualitative studies [11,27,28,29,30], funded by the German Health Care Innovation Fund to improve integrated and innovative care in Germany [31]. 

The objective of this part of the cross-sectional study was to explore the wide range of psychosocial stress and mental health problems of parents and their infants and toddlers. 

## 2. Materials and Methods

### 2.1. Study Design and Setting

The cross-sectional data presented here come from the SKKIPPI cohort study, a prospective observational population-based study. The study was conducted in three urban regions of northern and eastern Germany (Berlin, Flensburg, and Leipzig). After inclusion in the study, we screened for parents with an elevated mental health risk and collected relevant characteristics using an online screening questionnaire (Supplementary Material S1). Participants with an elevated mental health risk were invited for further assessments; details on these further assessments and the entire study were described previously [26]. The results of the in-depth psychiatric diagnostic interviews as well as identified risk factors will be presented elsewhere as they are too extensive to be reported here and would exceed the scope of this publication. The analysis presented in this paper contains only data collected with the above-mentioned online screening questionnaire, constituting the cross-sectional part of the SKKIPPI study. This article is reported in accordance with the ‘Strengthening the reporting of observational studies in epidemiology’ (STROBE) Statement checklist: cohort, case-control, and cross-sectional studies (combined) [32].

### 2.2. Study Population

A random sample of 30,000 addresses of families with children aged up to 12 months (at the time the sample was drawn) was provided by the residents’ registration offices in Berlin, Leipzig, and Flensburg. The children’s parents received at least one invitation letter to participate in this study. Invitation letters were sent to the registered mother but included two access codes, so both parents were able to participate in the first step of the study. Mothers who gave birth to more than one child within the 12-month sampling period (i.e., multiple births or two children in one year) received only one invitation letter. If no mother was registered, the person entitled to custody received the invitation letter. Other caregivers were able to complete the screening online questionnaire indicating their caregiver status, but only data from biological or adoptive parents were included for analysis. Inclusion criteria stated that parents had to be at least 18 years of age and able to complete the screening online questionnaire in the German, English, Turkish, or Arabic language. Written or online informed consent was obtained from all participants prior to study inclusion.

### 2.3. Data Collection

There are a variety of instruments used to identify psychosocial stress and occurrence of mental disorders in mothers and parents in the postpartum period. As these instruments are specific to the detection of certain illnesses or social problems, we developed a new instrument in order to obtain a broad overview of possible stress situations without being too complex and time-consuming. In addition, the threshold for study participation was kept low to avoid selection bias and to allow for participation of parents who are already very strained in the postnatal phase.

In the newly developed online screening questionnaire, the following data were collected, covering the four common domains of stress factors: parent, family, parent–child interaction, and child [33]. Sociodemographic characteristics, perinatal stressors and characteristics (e.g., planned pregnancy, multiple pregnancies, or complications during pregnancy), individual parental stressors (e.g., partnership/professional/financial problems or possible parental childhood trauma), and mental health problems in the parent and the child. Possible parental childhood trauma was assessed with the following single question “Have there been any severe, negative experiences in your childhood that you have not overcome to date?”. Data on mental health problems included questions on lifetime mental health disorders and current mental health problems. Current mental health problems were assessed using questions from the Patient Health Questionnaire (PHQ) 4 [34] and questions on obsessive–compulsive thoughts/acts from the Zohar-Fineberg Obsessive Compulsive Screen [35], as well as questions regarding alcohol/drug abuse [36] and current regulatory problems of the child [37]. The PHQ 4 is subdivided into two parts: the PHQ 2 (two-item depression scale) and the Generalized Anxiety Disorder (GAD) 2 (two-item anxiety scale) [34].

The online screening questionnaire (Supplementary Material S1) was newly developed by the study team, discussed with various experts, and piloted. We developed a scoring system with points ranging from 0 to 5 for each of the 40 questions and a cut-off of 5 points indicating an elevated mental health risk. The cut-off of the screening questionnaire was adjusted early in the study to 10 points because it was found that too many mothers without evidence of a mental health disorder in the further assessments of the study were identified as at risk by the online screening questionnaire. Data from the questionnaire were screened for plausibility and completeness. Answers in the questionnaire were validated at entry. User input that did not match a given data type or range was dismissed, and the user was prompted to answer the question again. It was not mandatory to answer questions, but when a question was skipped, the user was asked once for confirmation. 

### 2.4. Statistical Analysis

The data of the online screening questionnaire were analyzed descriptively. Results are presented in the form of means and standard deviations for continuous data, and frequencies and percentages for categorical data. The available data were used for each analysis, with missing data not being replaced. All statistical analyses were performed using SPSS Version IBM SPSS Statistics 26.

## 3. Results

### 3.1. Participants

**Figure 1:**
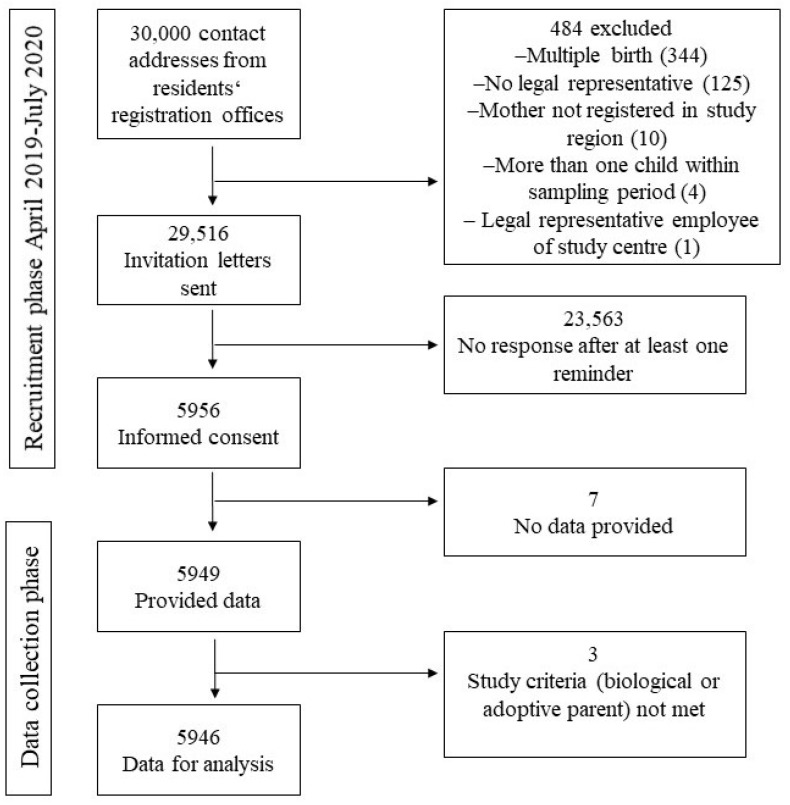
Details of the recruitment and data collection phase of the population-based cohort study in three cities.

Until the end of the recruitment period (March 2019–July 2020), 29,516 invitation letters were sent out, and at least one additional reminder letter was sent if the first letter was unanswered. A total of 484 parents received no letter, and the specific reasons for exclusion can be found in Figure 1. Nearly 99% of all parents filled out the screening online questionnaire completely. However, we also included data from questionnaires that were not completely filled out. Of all participants, 4981 (83.8%) indicated being biological mothers, three were adoptive mothers (0.1%), and 962 (16.2%) stated being biological fathers. No adoptive father participated in the study. In total, either the mother, the father, or both parents of 5151 children participated in the study (17.5%). Over 95% of the study parents participated prior to the 16 March 2020, the date when lockdowns and other measures related to the SARS-CoV-2 pandemic started in Germany.

### 3.2. Sociodemographic Characteristics

Most parents (mothers 74%, fathers 67%) were between 30 and 39 years of age (Table 1). Approximately 80% of them were born in Germany. Nearly 85% had a high educational level according to the International Standard Classification of Education (ISCED) categorization. Children ranged in age from 6 to 29 months (m = 14.6 months ± 3.0).

### 3.3. Perinatal Stressors and Characteristics

Approximately three quarters of both mothers and fathers indicated that the pregnancy was planned and the timing was suitable, in contrast to 5% of mothers and 3% of fathers who stated that the pregnancy was not planned and the timing not suitable (Supplementary Material S2). Three percent of the mothers delivered twins, and 7% delivered premature babies. Nearly one quarter of the babies were delivered by Caesarean birth.

### 3.4. Individual Parental Stressors

**Figure 2:**
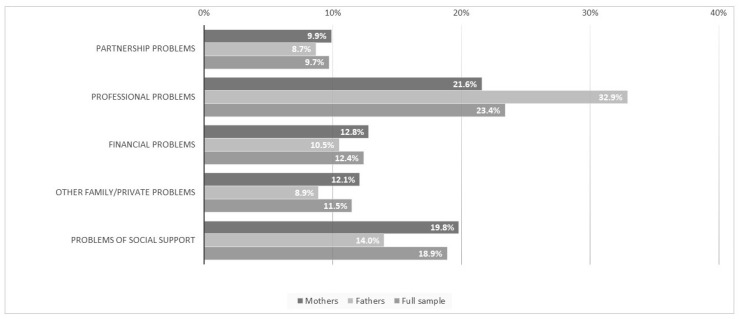
Various self-reported parental stressors (answers: “rather more” or “strong/very strong” combined).

One third of the fathers and 22% of the mothers reported to have rather more or strong/very strong professional problems (Figure 2). Severe, negative experiences in childhood that have not yet been overcome were disclosed in 22% of the mothers and 16% of the fathers (Table 2). In terms of digital media use, 16% of both parents indicated feeling stressed when their child sought attention while the parents were using their smartphone/tablet.

### 3.5. Mental Health Problems of the Parents and the Child

The lifetime occurrence of depression was 15% for mothers and approximately 7% for fathers, and anxiety disorders approached 6% for mothers and 3% for fathers (Table 3). Nearly one tenth of the mothers and fathers scored 3 or higher in the PHQ 2 Questionnaire (depressive symptoms within the PHQ 4) and 11% and 7%, respectively, in the GAD-2 Questionnaire (anxiety symptoms within the PHQ 4). Twenty-one percent and 15%, respectively, reported obsessive–compulsive thoughts, and 20% and 12% respectively reported mood swings and difficulties in controlling feelings. Between two and five percent of the parents reported that their child exhibited symptoms of one of the indicated regulatory problems at the time of survey (Table 4).

## 4. Discussion

### 4.1. Main Findings

Results from the cross-sectional data of the SKKIPPI cohort study showed that parents of children in the first years after birth were facing a variety of considerable psychosocial stressors. Obsessive–compulsive thoughts, mood swings, and difficulties in controlling feelings and depressive and anxiety symptoms were the most frequently reported mental health problems by both parents. At least one regulatory problem of the child was reported by approximately 5% of the parents. The use of the newly developed online screening questionnaire turned out to be feasible.

### 4.2. Comparison with Other Studies

Parents of nearly 18% of the children drawn from the residents’ registration offices in Berlin, Leipzig, and Flensburg participated in this cross-sectional first step of the study. During preparation of the study, a response rate of 20% was estimated [26], and the response rate achieved (17.5%) was only slightly below and corresponds to response rates from other epidemiological studies in Germany in previous years [38,39]. Nearly 99% of the consented participants also finished the questionnaire, so completeness of the data is very high. Given the fact that the invitation letter was primarily addressing the mother, the high number of mothers in the study sample (85%) is not surprising. To get a broader insight into the specific problems that fathers have in the early childhood period, future studies should address the fathers more comprehensively. As the majority of parents completed the online questionnaire before the restrictions and lockdowns due to the SARS-CoV-2 pandemic were implemented in Germany (95%), we assume that the results of this part of the study were not significantly influenced by the pandemic.

Our age range of 30–39 years for most participants corresponds to the average age of first-time parents in Germany. In 2020, mothers were on average 30 years old and fathers 33 years old at the time of birth of their first child [40]. It is striking that the number of single parents in our sample was relatively low (5% for both parents). In 2019, the proportion of single mothers or fathers among all families in Germany was 18.6% [41]. One reason for this difference could be the fact that our sample included only parents with very small children. It is likely that many parents separate a little later and not so soon after birth. The above-mentioned prevalence for Germany was assessed for all families with minor children and not only very small children. Another possible reason could be that single parents with small children have less time to participate in this kind of study.

Concerning adverse perinatal stressors, an unwanted/unplanned pregnancy is a known risk factor for postpartum depression [22]. In our questionnaire, we asked not only if the pregnancy was planned, but also if it was a suitable time. More than one fifth of the parents in our study reported that the pregnancy was not planned, a prevalence that has also been assessed by Lorenz et al. in another German sample [25]. Unfortunately, we do not know whether parents having ambivalent or adverse feelings about being pregnant had a higher or lower participation rate in this study. While more than 93% of the parents indicated that their child is or was breastfed at least to some extent, we did not ask how long they were breastfeeding for. There may be some mothers who only breastfed for a short period. We did not ask specific questions to ascertain whether breastfeeding was seen more as a stressful or positive experience for the parents. The same is true for other perinatal characteristics like childbirth setting, delivery procedure, or being a parent for the first time. This was a deliberate decision to ensure that the questionnaire did not become too long or too complex, leading to respondents dropping out of the survey. 

Parents in our study reported being confronted with a variety of individual stressors. We found the highest percentages for both mothers and fathers in the categories of professional problems and problems concerning lack of social support. The latter stressor is a well-known risk factor that has been investigated in various studies on postnatal depression [22,24]. Work-related or professional stress as a determinant for mental health problems has been assessed in various studies in men and women in the general population [42,43], but it seems to be less often considered in the postnatal setting and should be further investigated. Severe, negative experiences during childhood have been shown to be a risk factor for pre- and postnatal depression [44,45]. Comparing our study with another study on psychosocial stress, parents in our study more often reported severe, negative experiences during childhood [25]. As this stressor was only assessed with one single question in our study, this result should be interpreted with caution. Within the further assessments of the SKKIPPI cohort study, participants get a more detailed questionnaire on this topic, so we may get better insight into this particular stressor. A recent review on research related to parent distraction with phones and mobile devices suggests its adverse impact on parenting sensitivity and behaviors [46], but most evidence comes from self-reporting and observations. More longitudinal data are needed in this area of research. In our study, around 16% of mothers and fathers feel stressed if their child seeks their attention while they are using their smartphone/tablet. As mobile phones have become an indispensable part of parents’ daily lives, this influence should be investigated more specifically in future studies.

Obsessive–compulsive symptoms are less well studied during the postnatal period compared to depressive or anxiety problems, but various studies showed that the postpartum period is a high-risk time for this kind of mental health problem in mothers and fathers [47,48]. In our sample, a large number of parents reported obsessive–compulsive thoughts, but not as much obsessive–compulsive acts. Obviously, we cannot deduce from these results that all parents indicating obsessive–compulsive thoughts had an obsessive–compulsive disorder, as these kinds of thoughts can also be part of a depressive symptomatology. However, the high number found should draw more attention to this kind of symptom. Within the further assessments of the SKKIPPI cohort study, participants underwent a comprehensive psychiatric diagnostic interview; more precise numbers for obsessive–compulsive disorders in our sample will be reported elsewhere. As for the occurrence of actual depressive symptoms, former studies from Germany reported lower numbers of depression in mothers in the first months after birth, ranging from 3% to 6% [19,49]; however, samples were not population-based but hospital-based. Data from a recent large population-based study in Germany (based on PHQ 8) showed similar rates to those of our study; however, they included both women and men with and without children and not only parents (women 10.8% versus men 7.6%, n = 254,510, age ≥ 15 years) [50]. Interestingly, the age range of 15–29 years in the above-mentioned study showed the highest prevalence rates (11.5% for both men and women together). For anxiety problems, previous data from Germany showed similar frequencies for mothers in the first months after birth (circa 11%) [19], but the sample was hospital-based, not population-based.

Parent reporting of subjectively perceived regulatory problems in the children of our sample were lower than in other German studies [15,17]. Some of our participants indicated in the questionnaire that their children have had regulatory problems in the very first months after birth, but not anymore at the time of the assessment. Another reason for this difference could be that we did not implement a specific questionnaire for this kind of problem in the online questionnaire but asked only for the parental perception of the child’s current behavior. Within the further assessments of the SKKIPPI cohort study, the presence of problems concerning crying, feeding, and sleeping are investigated in more detail using the specific questionnaire developed by Groß and colleagues [37].

### 4.3. Strengths and Limitations

One of the strengths of our study is the large, population-based sample, while previous studies are often based on hospital or other routine healthcare related data. The Coronababy study used a comparable survey method for psychosocial stress in young parents, but it used a different field approach via pediatricians and also focused on stress during the SARS-CoV-2 pandemic [21]. We developed a short questionnaire in simple language assessing all kinds of parental stressors and translated it into several languages. We assessed not only typical maternal stressors but also paternal and child-related stressors.

However, several limitations must be discussed. Firstly, even if we recruited a population-based sample, it is not representative of Germany, since we recruited participants in only three regions of Germany, which are mostly urban. There is a need to get more data from rural regions where stressors can also be distinct from psychosocial and other stressors in urban regions and where family ties may be higher. The reason for choosing these specific regions was the location of the respective partner institutions of the overall SKKIPPI study project. Secondly, we could not assess specific data on the non-respondents of the study, so potential differences in the characteristics between responders and non-respondents are unknown. The educational level of the participants was very high (85% belonged to the highest group). This is a common finding in epidemiological studies and reflects a general underrepresentation of people with middle and low education levels [51,52]. Therefore, our goal to reach more people with lower education levels by keeping the questionnaire short and in simple language was not sufficiently achieved. Future studies should therefore try to implement specifically adapted strategies to recruit more people with lower educational levels, e.g., through more targeted sampling. Thirdly, we cannot exclude that social expectations may have played a role in answering some of the more personal questions. For example, in answering the questions if the pregnancy was planned, if the child was breastfed, or how close the relationship to the child is. However, we tried to reduce the social desirability bias by collecting data via online questionnaire and not by face-to-face interview. Fourthly, we collected data on the lifetime occurrence but not on the beginning of the mental health problems; thus, we were not able to assess their presence during pregnancy with this cross-sectional study aspect. The reason for this was based on considerations that such prenatal information may not be validly assessed retrospectively, and answers may be biased.

## 5. Conclusions

Our data analyses of the cross-sectional study part of a large German population-based cohort study indicated that a substantial proportion of parents were burdened by psychosocial problems and showed indications of mental health problems during this period. Based on these data, the availability of early preventive and low-threshold support measures in the health and social care system should be adapted to the needs of new parents. Future research efforts should include follow-up analyses of this cohort and further attempts to include more fathers, parents with lower socioeconomic status, and families with migration backgrounds in population-based studies. The newly developed questionnaire proved to be well-suited in terms of its length and interview effort to obtain an overview of the wide range of psychosocial stress experienced by parents in the postnatal period.

## Figures and Tables

**Table 1:** Sociodemographic characteristics of the parents (adoptive mothers included, n (%), ISCED = International Standard Classification of Education) in the population-based study from the SKKIPPI project.

	Mothers n (%)	Fathers n (%)	Full Sample n (%)
Study site	4984	962	5946
– Berlin	3963 (79.5)	745 (77.4)	4708 (79.2)
– Flensburg	67 (1.3)	14 (1.5)	81 (1.4)
– Leipzig	813 (16.3)	171 (17.8)	984 (16.5)
– Other region	141 (2.8)	32 (3.3)	173 (2.9)
Age group	4984	962	5946
– ≤29	778 (15.6)	88 (9.1)	866 (14.6)
– 30–39	3708 (74.4)	649 (67.5)	4357 (73.3)
– 40–49	497 (10.0)	203 (21.1)	700 (11.8)
– ≥50	1 (0.0)	22 (2.3)	23 (0.4)
Country of birth	4946	936	5882
– Germany	4190 (84.7)	786 (84.0)	4976 (84.6)
– Other country	756 (15.3)	150 (16.0)	906 (15.4)
Native language	4945	935	5880
– German	4232 (85.6)	796 (85.1)	5028 (85.5)
German language skills (if not native German, self-assessment)	711	139	850
– Very good/good	557 (78.3)	99 (71.2)	656 (77.2)
– Average	100 (14.1)	20 (14.4)	120 (14.1)
– Bad	54 (7.6)	20 (14.4)	74 (8.7)
Educational level	4944	935	5879
– Low (ISCED 1)	24 (0.5)	7 (0.7)	31 (0.5)
– Middle (ISCED 2)	695 (14.1)	127 (13.6)	822 (14.0)
– High (ISCED 3)	4196 (84.9)	796 (85.1)	4992 (84.9)
– Unknown	29 (0.6)	5 (0.5)	34 (0.6)
Current partner/relationship	4970	950	5920
– Yes	4749 (95.6)	947 (99.7)	5696 (96.2)
Single parent	4943	935	5878
– Yes	289 (5.8)	7 (0.7)	296 (5.0)
Number of children < 18 y in household (including index child)	4945	934	5879
– 0	111 (2.2)	19 (2.0)	130 (2.2)
– 1	2640 (53.4)	563 (60.3)	3203 (54.5)
– 2	1692 (34.2)	265 (28.4)	1957 (33.3)
– 3 or more	502 (10.2)	87 (9.3)	589 (10.0)
Receiving government benefit payments	4940	936	5885
– Yes	653 (13.2)	83 (8.9)	736 (12.5)
Support through early intervention programs	4943	935	5878
– Yes	727 (14.7)	156 (16.7)	883 (15.0)

**Table 2:** Individual parental stressors (adoptive mothers included, n (%)) in the population-based study from the SKKIPPI project.

	Mothers n (%)	Fathers n (%)	Full Sample n (%)
Index child diagnosed with a serious illness or disability after birth or in the first few months of life	4977	954	5931
– Yes	180 (3.6)	30 (3.1)	210 (3.5)
Other child in household with a serious illness or disability	4940	935	5875
– Yes	111 (2.2)	28 (3.0)	139 (2.4)
Relationship with the child (‘I feel close to my child …’)	4972	950	5922
– Always	3918 (78.8)	685 (72.1)	4603 (77.7)
– Very often	1000 (20.1)	249 (26.2)	1249 (21.1)
– Sometimes	48 (1.0)	15 (1.6)	63 (1.1)
– Rarely	6 (0.1)	1 (0.1)	7 (0.1)
– Never	0 (0.0)	0 (0.0)	0 (0.0)
Severe, negative experiences in own childhood	4944	938	5882
– Yes	1064 (21.5)	150 (16.0)	1214 (20.6)
Excessive preoccupation with diet and weight	4950	938	5888
– Yes	705 (14.2)	66 (7.0)	771 (13.1)
Current burdensome (chronic) illness	4947	936	5883
– Yes	377 (7.6)	80 (8.5)	457 (7.8)
Use of digital media: ‘I feel stressed if my child seeks my attention while I am using my smartphone/tablet.’	4968	948	5916
– Yes	785 (15.8)	153 (16.1)	938 (15.9)
Use of digital media: ‘When I feed or play with my child, I use my smartphone/tablet …’	4972	950	5922
– Generally not at all	1411 (28.4)	302 (31.8)	1713 (28.9)
– Rarely	2119 (42.6)	396 (41.7)	2515 (42.5)
– Sometimes	1253 (25.2)	224 (23.6)	1477 (24.9)
– Often	189 (3.8)	28 (2.9)	217 (3.7)

**Table 3:** Parental mental health problems (adoptive mothers included, n (%), PHQ = Patient Health Questionnaire, GAD = Generalized Anxiety Disorder) in the population-based study from the SKKIPPI project.

	Mothers n (%)	Fathers n (%)	Full Sample n (%)
Occurrence of parental mental health disorders (lifetime)	4940	935	5875
– None	3886 (78.7)	825 (88.2)	4711 (80.2)
– Diagnosis	4951	937	5888
– Depression	738 (14.9)	69 (7.4)	807 (13.7)
– Anxiety disorder	282 (5.7)	31 (3.3)	313 (5.3)
– Obsessive–compulsive disorder	35 (0.7)	7 (0.7)	42 (0.7)
– Psychosis	16 (0.3)	1 (0.1)	17 (0.3)
Occurrence of parental mental health problems (current situation)			
Depressive Symptoms (PHQ-2)	4955	944	5899
– Score ≥ 3	458 (9.2)	84 (8.9)	542 (9.2)
Anxiety symptoms (GAD-2)	4953	944	5897
– Score ≥ 3	537 (10.8)	70 (7.4)	607 (10.3)
Alcohol problems	4951	938	5889
– Yes	15 (0.3)	9 (1.0)	24 (0.4)
Drug problems	4950	937	5887
– Yes	9 (0.2)	5 (0.5)	14 (0.2)
Obsessive–compulsive thoughts	4948	938	5886
– Yes	1054 (21.3)	145 (15.5)	1199 (20.4)
Obsessive–compulsive acts	4951	938	5889
– Yes	271 (5.5)	60 (6.4)	331 (5.6)
Mood swings and difficulties in controlling feelings	4946	935	5881
– Yes	982 (19.9)	110 (11.8)	1092 (18.6)

**Table 4:** Parental self-assessment of regulatory problems of children (adoptive mothers included, n (%)) in the population-based study from the SKKIPPI project.

Regulatory Problem	Mothers n (%)	Fathers n (%)	Full Sample n (%)
Feeding problems	4972	945	5917
– Yes	71 (1.4)	20 (2.1)	91 (1.5)
Excessive crying	4972	950	5922
– Yes	151 (3.0)	20 (2.1)	171 (2.9)
Difficulties in falling asleep	4973	950	5923
– Yes	171 (3.4)	45 (4.7)	216 (3.6)
Difficulties in maintaining sleep	4973	950	5923
– Yes	246 (4.9)	59 (6.2)	305 (5.1)

## Data Availability

Data will be available from the authors upon reasonable request, with restrictions regarding scientific purpose and data protection.

## Supplementary Materials

The following supporting information can be downloaded at https://www.mdpi.com/article/10.3390/children11080920/s1, Supplementary Material S1: Screening tool.pdf, Supplementary Material S2: Stressors.pdf.
