# Supplementary material for: Assessment of Psychosocial Stress and Mental Health Disorders in Parents and Their Children in Early Childhood: Cross-Sectional Results from the SKKIPPI Cohort Study
Source: Children (Basel). 2024 Jul 30;11(8):920. doi: 10.3390/children11080920 (PMC11352251; doi:10.3390/children11080920)
Supplement: Supplementary file 1 [file children-11-00920-s001.zip › Supplementary material S1_Screening tool.pdf]

A screening instrument for psychosocial distress among parents and their children during their first year of life

The first months after birth are an exciting and happy time for many parents. But asides from the many beautiful moments, there are times for many parents where they feel stressed or overburdened. Please allow about 10-15 minutes to fill out the questionnaire. If you feel unsure when answering some of these questions, please always select the answer that applies to you **the most**.

**Date**	☐☐ ☐☐ ☐☐☐☐	
**1. What is your relation to the child?**	I am …<br>☐ The mother<br>☐ The father<br>☐ The adoptive mother<br>☐ The adoptive father	☐ The grandmother<br>☐ The grandfather<br>☐ The foster mother<br>☐ The foster father<br>☐ Other<br>__________________
**2. Does the child live with you?**	☐ Yes<br>☐ No<br><br>If no: Who does the child live with the most?<br>With …<br>☐ The mother<br>☐ The father<br>☐ The adoptive mother<br>☐ The adoptive father	☐ The grandmother<br>☐ The grandfather<br>☐ The foster mother<br>☐ The foster father<br>☐ other<br>__________________

In the following questions we would like to ask you about the pregnancy, the birth and the first months of your child's life.

**3. Was the pregnancy a multiple pregnancy?**	☐ No<br>☐ Yes, twins<br>☐ Yes, triplets or more
**4. Did you have a preterm delivery?**	☐ Yes<br>☐ No
**If yes: How many weeks before the calculated due date was your child born?**	☐☐ Weeks
**5. Did you have one or more complications during pregnancy?**<br><br>*(Multiple answers possible)*	☐ none<br>☐ strong bleeding<br>☐ diabetes during pregnancy<br>☐ preeclampsia (elevated blood pressure, protein in the urine, water retention)<br>☐ other
**6. Some pregnancies are planned others are not planned. Some births fall onto a suitable point in time your life, others not as much. What was it like for you?**<br>**Did the birth of your child fall onto a suitable point in time in your life?**	☐ Planned and suitable time<br>☐ Not planned, but suitable time<br>☐ Planned, but not a suitable time<br>☐ Not planned and not a suitable time
**7. Where was your child born?**	☐ Hospital<br>☐ birth house<br>☐ at home<br>☐ other
**8. How was the child delivered?**	☐ Normal (vaginal)<br>☐ Delivery with forceps or suction cup<br>☐ Planned C-Section<br>☐ Unplanned C-Section (emergency C-section)
**9. It this child your first child?**	☐ Yes<br>☐ No
**10. Was the child diagnosed with a serious illness or disability after birth or in the first few months of life?**	☐ Yes<br>☐ No<br>If yes, which one?<br>__________<br>__________

**11. Some children are breastfed, others are not. Which statement applies to your child?**	☐ Yes, my child is still being breastfed<br>☐ Yes, my child was breastfed, but not anymore<br>☐ No, my child was not breastfed

**If yes, how long was your child breastfed <u>in total</u>? About**	☐☐	months

**12. How would you describe your relationship to your child?**	Always	very often	Sometimes	Rarely	Never
**I feel close to my child …**	☐	☐	☐	☐	☐

Every baby has good and bad days. For the following questions please consider what applies to you and your baby <u>on most days</u>. Please think about a typical week recently.

(By typical week, we mean weeks that reflect your regular everyday life without any special occurrences. Non-typical weeks are weeks in which your regular everyday life is disrupted for several days, e.g. due to holidays/vacation, illness or rare occurrences or events.)

**13. Is feeding or breastfeeding normally easy for you to master?**	☐ Yes<br>☐ No<br><br>If no, do you still normally manage to motivate your child to eat/drink in those situations?<br>☐ Yes<br>☐ Sometimes<br>☐ No
**14. Does your child cry excessively?**	☐ Yes<br>☐ No<br><br>If yes, do you nevertheless manage to calm your child?<br>☐ Yes<br>☐ Sometimes<br>☐ No
**15. Does your child generally fall asleep easily?**	☐ Yes<br>☐ No<br><br>If no, do you nevertheless manage to help your child fall asleep? E.g. by feeding, caressing, cradling, carrying, singing or other sleeping aides?<br>☐ Yes

	☐ Sometimes<br>☐ No
**16. In your opinion, does your child wake up overly frequently during the night?**	☐ Yes<br>☐ No<br><br>If yes, do you manage to get your child to fall back asleep in those situations? E.g. by feeding, caressing, cradling, carrying, singing or using other sleeping aides?<br>☐ Yes<br>☐ Sometimes<br>☐ No

**17. Most parents use digital devices in their everyday lives (Smartphone, Tablet etc.). What applies to you?**	Often	Sometimes	Rarely	Generally not at all
**When I feed or play with my child, I use my smartphone/tablet…**	☐	☐	☐	☐

**18. I feel stressed if my child seeks my attention while I am using my smart phone/tablet.**	☐ Yes<br>☐ No

The birth of a child poses a big challenge that can be accompanied by stress or burdens for the parents or main reference persons of the child. You may find some of the following questions very personal. We nevertheless hope that you will still answer all of the questions if possible. Please remember, there are no right or wrong answers.

**19. Are you in a committed partnership/relationship?**	☐ Yes<br>☐ No<br><br>If yes, do you live with your partner?<br>☐ Yes, permanently<br>☐ Yes, but not permanently (e.g. only on weekends)<br>☐ No

	Not at all	A little	Rather more	Strongly or very strongly
(Only for those with a partner)<br>**20. Do you suffer from difficulties/conflicts with your partner?**	☐	☐	☐	☐
**21. Do you suffer from any job/career related stress (time constraints, excessive demands, conflicts, worries**	☐	☐	☐	☐

**regarding your employment, dissatisfaction with your work)?**				
**Do you suffer from a lack of support in your everyday life and in the care of your child?**	☐	☐	☐	☐
**Do you suffer from other family related or personal stress or conflict (not in relation to your partner)?**	☐	☐	☐	☐

In the first few months in the life of a child, your own wellbeing can be more or less compromised.

**22. <u>Over the last 2 weeks</u>, how often have you been bothered by the following problems?**	**Not at all**	**Several days**	**More than half the days**	**Nearly every day**
**Little interest or pleasure in doing things**	☐	☐	☐	☐
**Feeling down, depressed or hopeless**	☐	☐	☐	☐
**Feeling nervous, anxious or on edge**	☐	☐	☐	☐
**Not being able to stop or control worrying**	☐	☐	☐	☐

Asides from birth there are other things that can burden you in life. Which one of the following apply to you?

**23.**	Yes	No
**Do you have agonising thoughts you would like to stop, but can't?**	☐	☐
**Do you have to repeat certain actions over and over again, even though they seem senseless to you (e.g. washing hands compulsively & excessively, constantly checking the stove or coffee machine)?**	☐	☐
**Have there been any severe, negative experiences in your childhood that you have not overcome to date?**	☐	☐
**Are you <u>overly</u> concerned with your diet and weight?**	☐	☐

**24. Have you <u>ever</u> had problems with alcohol?**	☐Yes		☐No	
	**Not at all**	**hardly**	**A litte**	**A lot**

**If yes, how much do you <u>currently</u> feel affected by these problems?**	☐	☐	☐	☐
**25. Have you <u>ever</u> had problems with drugs?**	☐ Yes		☐ No	
	**Not at all**	**Hardly**	**A little**	**A lot**
**If yes, how much do you <u>currently</u> feel affected by these problems?**	☐	☐	☐	☐

**26. Do you have problems in controlling your feelings or do you suffer from erratic mood swings?**	☐ Yes<br>☐ No
**27. Have you ever <u>been</u> diagnosed with one of the following mental disorders?**<br><br>*(Multiple answers possible)*	☐ Depression<br>☐ Anxiety disorder<br>☐ Psychosis<br>☐ Obsessive compulsive disorder<br>☐ Other psychological disorders, i.e.: ____________<br>☐ none
**28. Do you <u>currently</u> suffer from a <u>chronic disease</u> that has had a strong negative impact on your everyday life?**	☐ Yes, i.e. ____________<br>☐ No
**29. Is there anything else that causes a burden or stress in your everyday life?**	☐ Yes, i.e. ____________<br>☐ No

Finally, we would like to ask you some questions about your life situation.

**30. How old are you?**	☐☐	Years old
**31. Where were you born?**	☐ Germany<br>☐ In another country: i.e.<br>____________	

**32. Is German your native language?**	☐ Yes<br>☐ No<br><br>If yes, do you have other native languages?<br>☐ No<br>☐ Yes, i.e.:<br>____________________	If German is not your native language, how good are your German skills in your opinion?<br>☐ Very good<br>☐ Good<br>☐ Intermediate<br>Poor

**33. What is your highest level of school you completed?**<br><br>*Please state the school level, country and number of school years completed in total*	School level/ country/ number of school years completed:<br>____________________<br>____________________<br>____________________<br>____________________

**34. How many adults (age 18 and older) live in your household (yourself excluded)?**	☐☐	Number of adults
**35. In total, how many children under the age of 18 live in your household?**<br>(This includes the child that this questionnaire refers to.)	☐☐	Number of children

**36. How old are these children?**	☐☐ Age of first child<br>☐☐ Age of second child<br>☐☐ Age of third child<br>☐☐ Age of fourth child<br><br>Ages of other children: __________<br>____________________<br>____________________
**37. Has one of the children in your household been diagnosed with a serious illness or disability?**	☐ Yes<br>☐ No<br><br>If yes, which illness or disability?<br>____________________

**38. Are you a single parent?**	☐ Yes<br>☐ No

**39. Have you or any other members of your household received one or more of the following German state payments in <u>the last 12 months</u>: unemployment benefits (type II/ Hartz IV), income support (Sozialgeld) or social welfare (Sozialhilfe)?**	☐ Yes<br>☐ No
**40. Have you and your child received any support through early help programs (Frühe Hilfen) <u>that came to your house</u> in the first months after birth?**<br>(e.g. a family midwife (support beyond regular post-partum care), several volunteer visits (e.g. from family patrons or support volunteers for parents) or measures from the youth welfare office).	☐ Yes<br>☐ No

41. Is there anything else you would like us to know?

Source of the Questions:
PBQ: Postpartum Bonding Questionnaire (Reck C, Klier C, Pabst K et al. The German version of the Postpartum Bonding Instrument: Psychometric properties and association with postpartum depression. Arch Womens Ment Health (2006) 9: 265.)
PHQ-4: Ultrakurzform des Gesundheitfragebogens für Patienten (Löwe B, Wahl I, Rose M et al.. A 4-item measure of depression and anxiety: validation and standardization of the Patient Health Questionnaire-4 (PHQ-4) in the general population. J Affect Disord. 2010 Apr;122(1-2):86-95.)
Z-FOCS: Zohar-Fineberg Obsessive Compulsive Screen (Fineberg NA, Roberts A (2001) Obsessive compulsive disorder: a twentyfirst century perspective. In Obsessive Compulsive Disorder: a Practical Guide (eds N. A. Fineberg, D. Marazziti & D. Stein), pp. 1-13. London: Martin Dunitz.)
KID 0-3: Nationale Prävalenzstudie zu psychosozialen Belastungen in der Frühen Kindheit (https://www.fruehehilfen.de/fileadmin/user_upload/fruehehilfen.de/pdf/Infas_Fragebogen_LebenssituationFamilien_20131113.pdf)
