# Supplementary material for: Assessment of Psychosocial Stress and Mental Health Disorders in Parents and Their Children in Early Childhood: Cross-Sectional Results from the SKKIPPI Cohort Study
Source: Children (Basel). 2024 Jul 30;11(8):920. doi: 10.3390/children11080920 (PMC11352251; doi:10.3390/children11080920)
Supplement: Supplementary file 1 [file children-11-00920-s001.zip › Supplementary material S2_Stressors.pdf]

Supplementary material 2:

Table: Perinatal stressors and characteristics (adoptive mothers excluded, n (%)) in the population-based study from the SKKIPPI project

	**Mothers n (%)**	**Fathers n (%)**	**Full sample n (%)**
**Pregnancy planned/suitable time**	4,975	955	5,930
– Planned and suitable time	3,567 (71.7)	707 (74.0)	4,274 (72.1)
– Not planned, but suitable time	838 (16.8)	160 (16.8)	998 (16.8)
– Planned, but not a suitable time	320 (6.4)	61 (6.4)	381 (6.4)
– Not planned and not a suitable time	250 (5.0)	27 (2.8)	277 (4.7)
**Multiple pregnancy**	4,977	960	5,937
– Twins	150 (3.0)	28 (2.9)	178 (3.0)
– Triplets or more	1 (0.0)	1 (0.1)	2 (0.0)
**Complications during pregnancy, (multiple answers possible)**	4,977	960	5,937
– Heavy bleeding	151 (3.0)	15 (1.6)	166 (2.8)
– Diabetes during pregnancy	360 (7.2)	62 (6.5)	422 (7.1)
– Preeclampsia	238 (4.8)	51 (5.3)	289 (4.9)
– Other complications	752 (15.1)	122 (12.7)	874 (14.7)
**Preterm delivery**	4,977	958	5,935
– Yes	344 (6.9)	74 (7.7)	418 (7.0)
**If yes:**			
– 3-7 weeks earlier	271 (78.8)	64 (86.5)	335 (80.1)
– >= 8 weeks earlier	71 (20.6)	10 (13.5)	81 (19.4)
– Unknown	2 (0.6)	0 (0.0)	2 (0.5)
**Childbirth setting**	4,976	954	5,930
– Hospital	4,643 (93.3)	888 (93.1)	5,531 (93.3)
– Birthing centre	231 (4.6)	48 (5.0)	279 (4.7)
– Home birth	97 (1.9)	17 (1.8)	114 (1.9)
– Other	5 (0.1)	1 (0.1)	6 (0.1)
**Delivery procedure**	4,976	954	5,930
– Normal (vaginal)	3,342 (67.2)	650 (68.1)	3,992 (67.3)
– Forceps or vacuum extraction	469 (9.4)	98 (10.3)	567 (9.6)
– Planned C-Section	493 (9.9)	81 (8.5)	574 (9.7)
– Unplanned C-Section (emergency C-section)	672 (13.5)	125 (13.1)	797 (13.4)
**First child**	4,975	954	5,929
– Yes	2,873 (57.7)	598 (62.7)	3,471 (58.5)
**Child breastfed**	4,969	951	5,920
– Is/was breastfed	4,632 (93.2)	894 (94.0)	5,526 (93.3)
– Never breastfed	337 (6.8)	57 (6.0)	394 (6.7)
